# Impact of Storage Temperature and Duration on Dimensional Stability and Mechanical Properties of 3D‐Printed Implant Surgical Guides

**DOI:** 10.1111/jerd.70198

**Published:** 2026-05-29

**Authors:** Sung‐Jin Kim, Koungjin Park, René Daher, Jin‐Soo Ahn, Vincent Fehmer, Jae‐Hyun Lee

**Affiliations:** ^1^ Department of Prosthodontics Seoul National University School of Dentistry Seoul Republic of Korea; ^2^ Division of Cariology and Endodontology University Clinics of Dental Medicine, University of Geneva Geneva Switzerland; ^3^ Department of Dental Biomaterials Science Seoul National University School of Dentistry Seoul Republic of Korea; ^4^ Dental Research Institute Seoul National University School of Dentistry Seoul Republic of Korea; ^5^ Division of Fixed Prosthodontics University Clinics of Dental Medicine, University of Geneva Geneva Switzerland

**Keywords:** computer‐assisted surgery, hardness, temperature, three‐dimensional printing, time factors

## Abstract

**Objective:**

This study aimed to evaluate the impact of storage temperature and duration on the dimensional stability and mechanical properties of 3D‐printed surgical guides.

**Materials and Methods:**

Sixty surgical guides were designed using two software programs (3Shape and SMOP) and printed with NextDent SG resin. Specimens were stored at 2°C, 20°C, and 30°C (*n* = 10). Scans were performed at baseline and after 2, 4, and 6 weeks to assess dimensional stability by using root mean square (RMS) deviation. Additionally, 30 bar‐shaped and 30 disk‐shaped specimens were printed (*n* = 10) to evaluate flexural strength, flexural modulus, and hardness after 6 weeks of storage at the respective temperatures.

**Results:**

Storage temperature had no significant effect on dimensional stability for 3Shape or SMOP. However, RMS values increased significantly from 2 to 6 weeks for both groups. In standardized mechanical specimens, storage temperature significantly affected flexural strength (*p* < 0.001) and flexural modulus (*p* = 0.018), but not Vickers hardness (*p* = 0.126). Flexural strength was highest at 30°C, while 2°C and 20°C did not differ significantly (*p* = 0.772). Flexural modulus was higher at 20°C than at 2°C (*p* = 0.038).

**Conclusions:**

Storage temperature did not affect dimensional stability or hardness but influenced flexural properties in standardized specimens. Time‐dependent dimensional changes were detected. Based on mechanical data, storage at 20°C or 30°C may be preferable to refrigeration for maintaining flexural performance.

**Clinical Significance:**

3D‐printed surgical guides demonstrated dimensional stability across varying storage temperatures, although time‐dependent deviations were detected. Based on standardized mechanical specimens, refrigeration may not provide mechanical benefits compared with room‐temperature storage. Therefore, room‐temperature storage may be preferable when delayed use is expected, even in warmer climates.

## 1 Introduction

Dental implants have been successfully used in clinical practice for the restoration of missing teeth and the improvement of masticatory function [1, 2]. For successful outcomes, accurate positioning of the implant is a prerequisite. The advancement of digital technology, particularly additive manufacturing techniques, has revolutionized implant dentistry by enabling the rapid and cost‐effective production of surgical guides [3]. The accuracy of these 3D‐printed surgical guides is critical, as it ensures precise implant placement, prevents damage to anatomical structures during surgery, and supports the long‐term postoperative stability of peri‐implant hard and soft tissues [4].

The accuracy of 3‐dimensional (3D)‐printed surgical guides is influenced by multiple factors throughout the digital workflow [5, 6]. Previous studies have evaluated how these factors, such as printer type, layer thickness, build orientation, storage, and sterilization, affect guide accuracy and internal surface fidelity [7, 8, 9, 10, 11, 12, 13, 14]. Clinically, guide accuracy is directly correlated with the deviation between the planned and actual implant positions [15, 16]. In this context, dimensional stability, particularly at the intaglio surface, which determines the seating on the supporting dentition, is paramount [14]. Any dimensional inaccuracy in this interface can compromise stabilization during guided drilling, thereby contributing to significant positional deviations.

In routine clinical practice, surgical guides are not always utilized immediately following fabrication. Due to logistical scheduling conflicts or patient‐related factors, surgery may be postponed, resulting in storage periods extending to several weeks. During this interval, polymer‐based materials are susceptible to continued post‐polymerization, stress relaxation, and physicochemical aging, which can induce alterations in both geometry and mechanical behavior [17]. Evidence regarding dimensional instability is well‐established not only for additively manufactured dental casts but also for conventional materials, such as pattern resins and polymerized prostheses, where storage significantly influences accuracy [17, 18, 19, 20, 21, 22, 23, 24]. Consistent with these observations, previous investigations have reported time‐dependent changes in the accuracy of 3D‐printed surgical guides [5, 25, 26]. Furthermore, the influence of environmental storage conditions, such as exposure to sunlight or darkness, on dimensional stability has also been documented [13]. However, comprehensive data regarding the effects of storage under various temperature conditions remain limited.

Despite the global variability in climatic conditions [27, 28], the impact of storage temperature on the dimensional stability of 3D‐printed surgical guides has not been systematically investigated. Indoor storage temperatures fluctuate based on seasonal and regional variations; in regions with high ambient temperatures or facilities lacking climate control, indoor temperatures may frequently approach 30°C [27, 28]. Most existing studies have focused on relatively short observation windows and have not explicitly examined temperature as an independent variable over longer durations [25, 26, 29]. Common surgical guide designs include the solid‐type (closed/full‐coverage) and the spaghetti‐type (open‐frame) configurations, which differ in surgical visibility, irrigation efficacy, and overall workflow handling, and the dimensional response to storage temperature may also differ across designs [30]. Therefore, this in vitro study aimed to assess the effects of storage temperature (2°C, 20°C, and 30°C) and storage duration (2, 4, and 6 weeks) on the dimensional stability of solid‐type and spaghetti‐type 3D‐printed surgical guides and the mechanical properties of the 3D‐printed resin for surgical guides.

## 2 Material and Methods

### 2.1 Study Design and Sample Size Calculation

**FIGURE 1:**
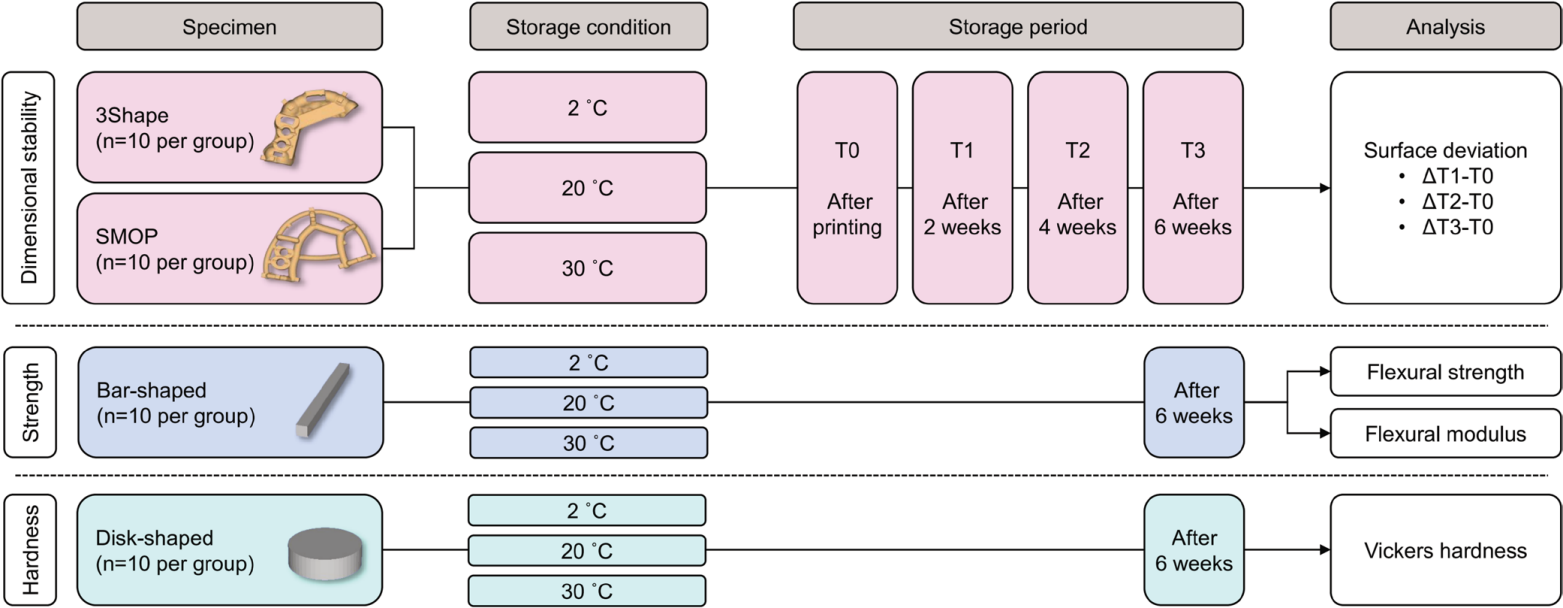
Schematic overview of study workflow illustrating specimen preparation, storage conditions, and analysis methods.

This in vitro study evaluated the 3D dimensional stability, flexural strength, and Vickers hardness of 3D‐printed implant surgical guides under various storage temperatures (Figure 1). Storage temperatures were selected to reflect diverse climatic conditions and weather patterns. Temperatures of 20°C and 30°C were chosen to represent standard indoor room temperatures in specific climatic zones. Additionally, storage at 2°C was included to evaluate the effects of refrigeration, which is a readily available storage method in clinical settings. A power analysis indicated that 10 specimens per group would be sufficient to achieve 80% power at a significance level of 0.05, under the assumption of a large effect size, which is in accordance with similar studies [25, 26, 29].

### 2.2 Surgical Guide Preparation

#### 2.2.1 Designing Surgical Guides

Two representative types of tooth‐supported surgical guides, named “solid‐type (3Shape)” and “spaghetti‐type (SMOP),” were selected for evaluation in this study. A commercial partially edentulous maxillary typodont model (E50‐523; Nissin Dental Products), missing teeth at the right maxillary second premolar and first molar positions, was scanned using a desktop scanner (Medit T710; Medit). The resulting data were exported as a standard tessellation language (STL) file and subsequently sent to two companies specializing in implant surgical guide design: (1) Solid‐type, OneGuide by Osstem Implant, utilizing 3Shape Implant Studio software (3Shape A/S); and (2) Spaghetti‐type, SMOP by Swissmeda AG utilizing SMOP software (Swissmeda AG). Two internal connection‐type bone‐level implants (TS III; Osstem Implant), with diameters of 4.0 and 4.5 mm for the second premolar and first molar, respectively, were simulated at depths of 4 mm.

**FIGURE 2:**
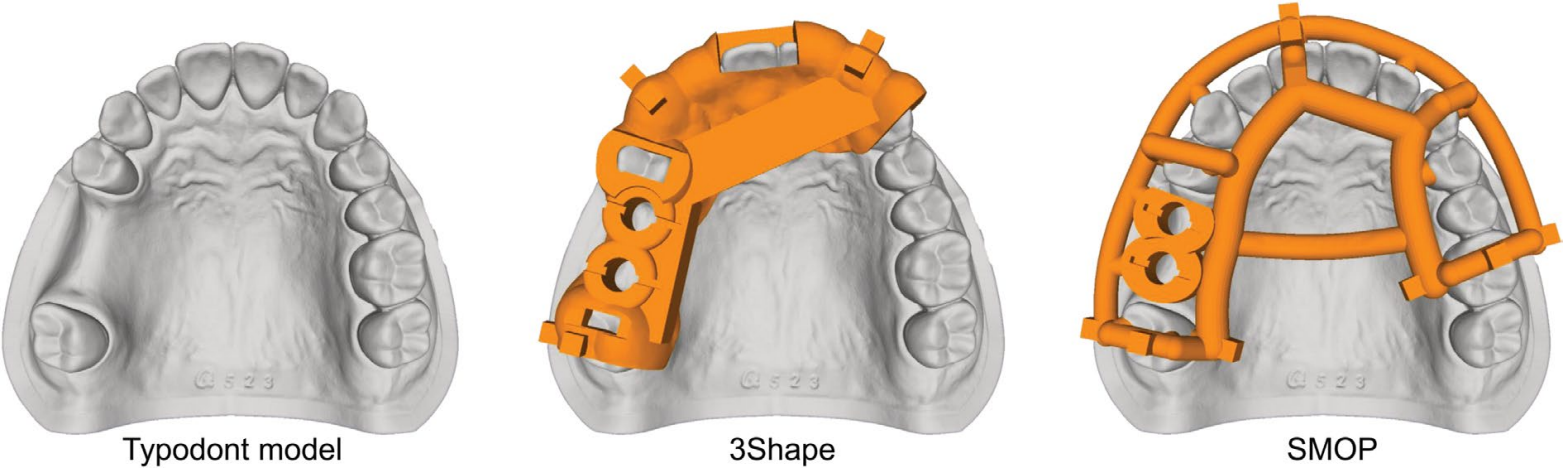
Computer‐aided design of implant surgical guides. (Left) Digitized dentiform model. (Middle) Full‐coverage “solid‐type” guide (3Shape). (Right) Open‐frame “spaghetti‐type” guide (SMOP).

Both types of surgical guides were designed by expert designers from each company, following their standard procedures commonly applied in clinical practice (Figure 2). The solid‐type surgical guide designed with 3Shape Implant Studio featured a rigid and structurally robust framework extending to the contralateral canine area, providing stability despite limited tooth‐surface coverage. In contrast, the spaghetti‐type surgical guide designed with SMOP software featured a minimalistic, open‐framework structure extending posteriorly to the contralateral molar to enhance stability. Both guide designs were exported as STL files and further processed using Meshmixer software, where cube‐shaped reference markers were added to their cameo surfaces to facilitate alignment during subsequent 3D superimposition analyses. The files were then re‐exported as STL files for 3D printing.

#### 2.2.2 3D‐Printing and Baseline Scanning

The design files were printed using a 3D printer (Asiga MAX UV; Asiga) with a 3D printing resin for surgical guides (NextDent SG; NextDent B.V.) at a layer thickness of 50 μm. The surgical guides were printed at 90° relative to the build platform, which was the orientation routinely used by the dental laboratory for this guide geometry to improve production efficiency and support placement. Because build orientation can influence the dimensional accuracy and mechanical behavior of additively manufactured surgical templates, the orientation was standardized within each specimen type. After printing, the guides were rinsed twice with 99% isopropyl alcohol (Yekyung Chemtech) for 5 min each using a vortex‐type cleaning system (Twin Tornado; Medifive). The guides were then post‐polymerized in an ultraviolet polymerization machine (Pure Pro; U‐DENT) for 10 min at 50°C, following the manufacturer's recommendations. Support structures were removed using a thin carbide bur, and the entire surface of each surgical guide was scanned with a desktop scanner (Medit T710; Medit). The scan data served as the baseline data (T0).

### 2.3 Storage Temperatures and Conditions

A total of 60 implant surgical guides were fabricated, comprising 30 3Shape designs and 30 SMOP designs. These guides were categorized into 3 subgroups based on storage temperature (*n* = 10 per group): 2°C, 20°C, and 30°C. The 20°C group served as the control, reflecting standard room temperature as defined by ISO 1:2022 [31]. The 30°C group simulated extreme summer conditions or regions with hot climates [27, 28], while the 2°C group represented refrigerated storage commonly employed in dental clinics. Each guide was individually sealed in a plastic bag. The 20°C and 30°C groups were stored in a constant temperature‐controlled incubator (FTLT‐MI; Sci Finetech), whereas the 2°C group was stored in a refrigerator (FR‐B153RW; Winia Aid). Both the incubator and refrigerator were situated in areas protected from direct sunlight.

### 2.4 Analyses of Dimensional Stability

Surgical guides were scanned at 2 weeks (T1), 4 weeks (T2), and 6 weeks (T3) during storage. Scanning was performed with the same desktop scanner (Medit T710; Medit) used for the baseline scan (T0). Between scanning sessions, the guides were returned to their designated storage conditions. Before each scanning session, the guides were transferred from their storage location to the scanning room and allowed to equilibrate at room temperature for at least 30 min. The scanning room temperature was approximately 20°C–22°C during the scanning procedures. This procedure was intended to reproduce a clinically relevant workflow, in which surgical guides are typically retrieved from storage and transferred to the operatory before surgery rather than used immediately after removal from storage. Prior to each scanning session, an antireflective powder (IP Scan‐spray; IP Division) was applied as a thin, uniform layer from a 3 cm distance to reduce surface reflectivity, following the manufacturer's instructions. Scan data were exported in STL file format.

To evaluate 3D dimensional stability, surface deviations on the intaglio surface were assessed. STL files from T0, T1, T2, and T3 were imported into 3D metrology software (Geomagic Control X, v2024.1; 3D Systems). The T0 scan of each guide was used as the reference dataset, and the corresponding T1, T2, and T3 scans were used as comparison datasets. Each comparison dataset was aligned to its corresponding T0 reference using cube‐shaped markers for initial alignment, followed by a best‐fit alignment procedure. Subsequent analyses focused exclusively on the intaglio surface. Root mean square (RMS) deviation values were calculated to quantify the dimensional change from T0 to each storage time point, and deviation color maps were generated for visualization.

### 2.5 Flexural Properties

Thirty bar‐shaped specimens (2 × 2 × 25 mm) were 3D printed at a 0° build orientation using the same material, layer thickness, and post‐processing protocol applied for the dimensional stability analysis. This orientation was selected to obtain straight flexural specimens with the long axis parallel to the build platform, so that the loading axis was perpendicular to the layer interfaces. These specimens were divided into 3 groups according to storage conditions: 2°C, 20°C, and 30°C (*n* = 10 per group) and stored for 6 weeks following the previously described procedure. A 3‐point bending test was conducted per ISO 10477:2020 guidelines using a universal testing machine (Model TW‐D102; Taewon Tech) [32]. Specimens were placed on two rounded supports with a span length of 20 mm, and a uniaxial load was applied at a crosshead speed of 1 mm/min until fracture. The maximum force before fracture was recorded, and the flexural strength and flexural modulus were calculated.

### 2.6 Vickers Hardness

Thirty disk‐shaped specimens (diameter, 10 mm; thickness, 3 mm) were 3D printed at a 90° build orientation using the same material, layer thickness, and fabrication process as described earlier. This orientation was selected to standardize support placement away from the hardness indentation surface and to maintain the same build orientation as the surgical guides. These specimens were assigned to three groups based on storage temperature: 2°C, 20°C, and 30°C (*n* = 10 per group), and stored for 6 weeks. Following storage, Vickers hardness was measured using a Vickers hardness tester (HM‐200; Mitutoyo) under a load of 0.05 kgf and a dwell time of 15 s. The Vickers hardness number (HV, kgf/mm^2^) for each specimen was calculated as the average of 5 measurements made from the specimen's surface.

### 2.7 Statistical Analysis

The normality of the data distribution was evaluated using the Kolmogorov–Smirnov test. For dimensional stability, a 3 (temperature) × 3 (time) repeated‐measures analysis of variance (ANOVA) was performed for each guide design separately. Sphericity was evaluated using Mauchly's test, and the Greenhouse–Geisser correction was applied when the sphericity assumption was violated. Pairwise comparisons of estimated marginal means were performed with Bonferroni adjustment. Direct statistical comparison between the 3Shape and SMOP guides was not performed due to inherent differences in their surface areas and design geometries. Regarding mechanical properties, flexural strength and Vickers hardness were analyzed using one‐way ANOVA followed by Tukey HSD post hoc test. For flexural modulus, because the normality assumption was not met, the Kruskal‐Wallis test was used, followed by Mann–Whitney *U* tests with Bonferroni correction. Effect sizes were reported as partial eta‐squared (*η* ^2^ *p*) for ANOVA effects, epsilon‐squared (*ε* ^2^) for the Kruskal–Wallis test, and rank‐biserial correlation (*r*) for Mann–Whitney *U* pairwise comparisons. Means are reported with standard deviation, and 95% confidence intervals (CIs) are reported for the marginal means and for the parametric mechanical outcomes; for nonparametric data, medians, interquartile ranges (IQRs), and bootstrap 95% CIs (10,000 resamples) are reported. Observed (post hoc) statistical power was computed at *α* = 0.05 for nonsignificant comparisons of primary interest. All statistical analyses were performed using SPSS Statistics (ver. 26; IBM), with the significance level set at *α* = 0.05.

## 3 Results

### 3.1 Dimensional Stability

**FIGURE 3:**
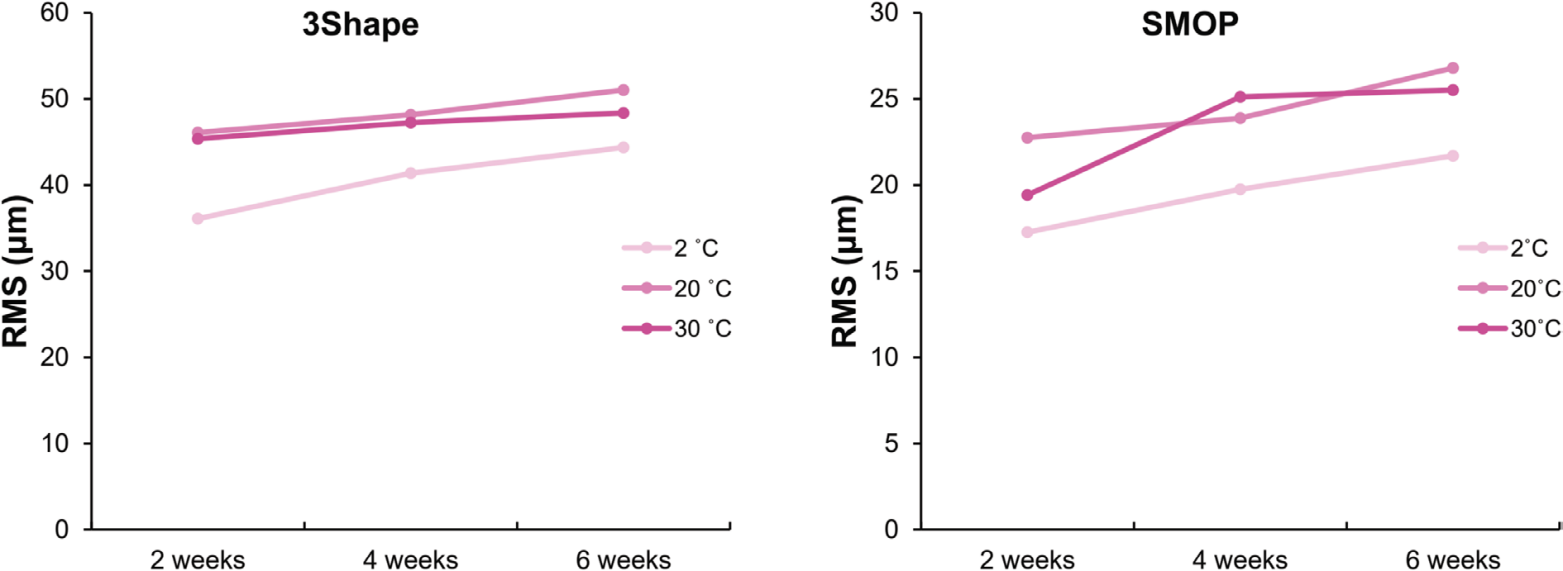
Dimensional stability of 3D‐printed surgical guides (3Shape group and SMOP group) expressed as root mean square deviation values (RMS; μm) from the baseline scan (T0) over storage time across different storage temperatures.

**FIGURE 4:**
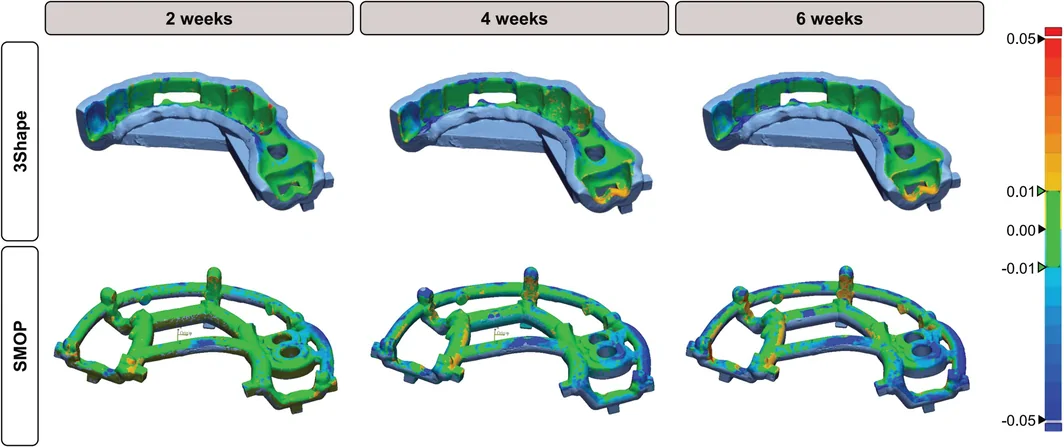
Representative color maps showing surface deviations of the intaglio surfaces across different storage durations (2, 4, and 6 weeks).

The RMS deviation values for the intaglio surfaces of the surgical guides are summarized in Table 1 and Figure 3, and the data distributions are summarized as boxplots in Figure S1. Representative color maps illustrating surface deviations are presented in Figure 4.

**TABLE 1:** Intaglio surface deviation (root mean square [RMS], μm) from baseline T0 scan according to storage temperature and storage duration.

Guide design	Storage duration (weeks)^†^	Storage temperature		
		2°C	20°C	30°C
3Shape	2ᵃ	36.10 ± 20.42	46.09 ± 23.01	45.37 ± 31.08
	4ᵃᵇ	41.39 ± 21.87	48.17 ± 28.21	47.24 ± 30.64
	6ᵇ	44.37 ± 25.56	51.04 ± 27.72	48.38 ± 31.86
SMOP	2ᵃ	17.26 ± 9.04	22.75 ± 5.20	19.42 ± 8.33
	4ᵇ	19.76 ± 5.93	23.88 ± 7.60	25.13 ± 8.16
	6ᵇ	21.70 ± 4.82	26.80 ± 6.98	25.52 ± 9.78

*Note:* Values represented as mean ± standard deviation (μm).

^**†**^ Different superscript lowercase letters indicate significant differences among storage durations within each guide design (*p* < 0.05). No significant differences among temperatures at each storage duration (*p* > 0.05). No direct statistical comparison between 3Shape and SMOP guides due to inherent differences in surface areas and design geometries.

For the 3Shape group, Mauchly test indicated that the sphericity assumption was violated (*W* = 0.677, *p* = 0.006), and the Greenhouse–Geisser correction was therefore applied. The main effect of storage temperature was not significant (*F*(2, 27) = 0.246, *p* = 0.784, *η* ^2^ *p* = 0.018; observed power = 0.085), and the Time × Temperature interaction was also not significant (*F*(3.02, 40.82) = 0.494, *p* = 0.690, *η* ^2^ *p* = 0.035; observed power = 0.142). RMS values increased significantly with storage duration (*F*(1.51, 40.82) = 5.539, *p* = 0.013, *η* ^2^ *p* = 0.170; observed power = 0.750). Bonferroni‐adjusted pairwise comparisons showed that the RMS at 6 weeks (47.93 μm; 95% CI [37.25, 58.61]) was significantly higher than at 2 weeks (42.52 μm; 95% CI [33.06, 51.98]; mean difference 5.41 μm, *p* = 0.030); Week 2 vs. Week 4 (mean difference 3.08 μm, *p* = 0.249) and Week 4 vs. Week 6 (mean difference 2.33 μm, *p* = 0.137) did not reach significance. Coefficients of variation in the 3Shape group ranged 49.9%–68.5% across conditions, and Levene test confirmed homogeneous error variances at all time points (*p* > 0.05). A Tukey outlier check identified no outliers in the 3Shape group and one outlier in the SMOP group at 2 weeks and 2°C. All 10 specimens per group were retained for analysis.

For the SMOP group, Mauchly test indicated that the sphericity assumption was met (*W* = 0.974, *p* = 0.710). Neither the main effect of storage temperature (*F* (2, 27) = 1.550, *p* = 0.231, *η* ^2^ *p* = 0.103; observed power = 0.300) nor the Time × Temperature interaction (*F*(4, 54) = 0.685, *p* = 0.606, *η* ^2^ *p* = 0.048; observed power = 0.208) was significant. A significant main effect of storage duration was identified (*F*(2, 54) = 8.970, *p* < 0.001, *η* ^2^ *p* = 0.249; observed power = 0.966). Bonferroni‐adjusted pairwise comparisons showed significant increases from Week 2 (19.81 μm; 95% CI [16.92, 22.70]) to Week 4 (22.92 μm; 95% CI [20.19, 25.65]; mean difference 3.11 μm, *p* = 0.043) and from Week 2 to Week 6 (24.67 μm; 95% CI [21.87, 27.47]; mean difference 4.86 μm, *p* = 0.001); the difference between Week 4 and Week 6 (mean difference 1.75 μm, *p* = 0.339) was not significant. Coefficients of variation in the SMOP group ranged 22.9%–52.4%.

### 3.2 Mechanical Properties

**FIGURE 5:**
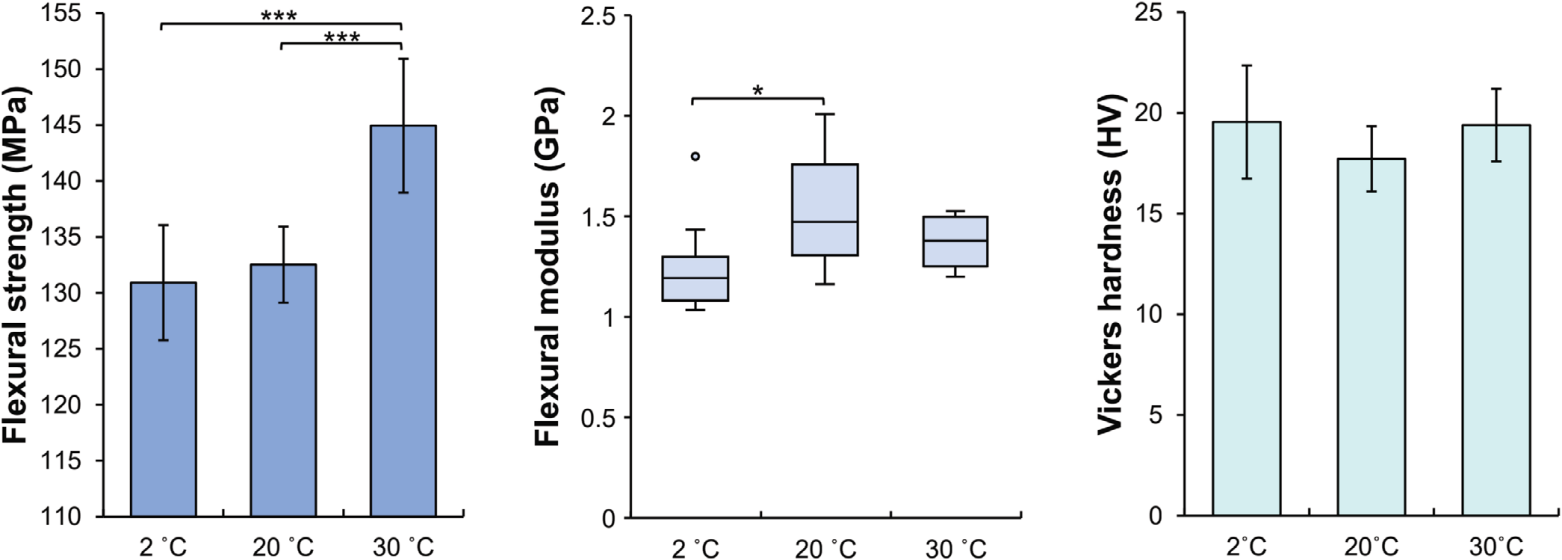
Mechanical properties (flexural strength, flexural modulus, and Vickers hardness) of 3D‐printed specimens after 6 weeks of storage at different temperatures (2°C, 20°C, and 30°C). Error bars for bar graphs represent standard deviation. *p < 0.05; ***p < 0.001.

The mechanical properties of the specimens after 6 weeks of storage, including flexural strength, flexural modulus, and Vickers hardness, are presented in Figure 5.

For flexural strength, one‐way ANOVA showed a significant effect of storage temperature (*F*(2, 27) = 21.54, *p* < 0.001, *η* ^2^ *p* = 0.615). Tukey HSD post hoc tests indicated that specimens stored at 30°C had the highest mean flexural strength (144.94 ± 6.30 MPa; 95% CI [140.43, 149.45]), which was significantly higher than those stored at 20°C (132.53 ± 3.59 MPa; 95% CI [129.96, 135.10]) and 2°C (130.91 ± 5.43 MPa; 95% CI [127.03, 134.79]) (both *p* < 0.001). The 2°C and 20°C groups did not differ significantly (*p* = 0.772).

For flexural modulus, the Kruskal–Wallis test was used because normality was violated in the 2°C group; the temperature effect was significant (*H* (2) = 8.00, *p* = 0.018, *ε* ^2^ = 0.222). Post hoc Mann–Whitney U tests with Bonferroni correction showed that the 20°C group (median 1.47 GPa, IQR 0.45; 95% CI [1.31, 1.75]) had a significantly higher modulus than the 2°C group (median 1.19 GPa, IQR 0.22; 95% CI [1.08, 1.33]; adjusted *p* = 0.038; rank‐biserial *r* = 0.66). The 30°C group (median 1.38 GPa, IQR 0.25; 95% CI [1.24, 1.49]) did not differ significantly from the 2°C (rank‐biserial *r* = 0.54) or 20°C (rank‐biserial *r* = 0.34) groups (both adjusted *p* > 0.05).

Vickers hardness was not significantly affected by storage temperature (*F* (2, 27) = 2.23, *p* = 0.126, *η* ^2^ *p* = 0.142; observed power = 0.454). The mean hardness values were 19.54 ± 2.81 HV (95% CI [17.53, 21.55]) for the 2°C group, 17.72 ± 1.62 HV (95% CI [16.56, 18.88]) for the 20°C group, and 19.39 ± 1.80 HV (95% CI [18.10, 20.68]) for the 30°C group.

## 4 Discussion

In this study, storage temperature did not significantly affect the intaglio surface dimensional stability of either surgical guide design, whereas storage duration significantly increased RMS deviation over the 6‐week period. Conversely, storage temperature significantly influenced flexural strength and flexural modulus after 6 weeks, although Vickers hardness remained unaffected.

Regarding dimensional stability, neither the 3Shape nor the SMOP guide showed a significant main effect of storage temperature or a temperature × duration interaction, indicating that within the tested range (2°C–30°C), temperature alone did not significantly alter intaglio surface trueness. This finding partially contrasts with previous reports describing significant temperature‐dependent dimensional changes in larger 3D‐printed objects, such as dental casts [18, 20]. However, detailed comparisons reveal consistent patterns. A previous study reported no significant difference in dimensional accuracy between dental models stored at 4°C and 25°C [18], which aligns with the lack of significant difference observed among temperature groups in the present study. Furthermore, it has been demonstrated that specimens stored at cold temperatures (4°C) exhibited relatively higher accuracy compared to those stored at higher temperatures [18, 20]. This trend is consistent with the present results, where the 2°C group showed the lowest absolute deviation values, although the difference was not statistically significant. A plausible explanation for this discrepancy in statistical significance is the structural difference between the objects; surgical guides have a considerably smaller overall volume and different geometry compared to solid dental casts. These characteristics likely limit the magnitude of polymerization‐related shrinkage, making the absolute dimensional changes too small to be detected as statistically significant within the tested temperature range.

The potential influence of thermal expansion during scanning should also be considered, as dental resins exhibit measurable coefficients of linear thermal expansion [33]. In the present study, all guides were equilibrated at room temperature for at least 30 min before scanning, which likely reduced this effect. However, because the surface temperature of each guide was not directly measured, residual thermal expansion effects cannot be completely excluded.

Storage duration significantly affected the RMS deviation of both guide types, with greater deviations observed at 6 weeks compared to 2 weeks. These findings align with previous studies reporting decreased dimensional stability of printed guides after extended storage periods of approximately 20–30 days [5, 25, 26]. Specifically, significant dimensional changes were observed in DLP‐printed guides over 20 days [25]. Notably, it has been reported that the accuracy and stability of additively manufactured resin surgical templates significantly decreased after 1 month of storage, whereas the metal templates evaluated in the same investigations remained dimensionally stable [5, 26]. This distinction highlights the specific susceptibility of resin materials to time‐dependent deformation compared to metals. Conversely, another study found no significant impact of storage time on intaglio surface accuracy over a shorter period of 14 days [29]. This discrepancy suggests that while dimensional changes may be negligible within the first 2 weeks, cumulative effects such as ongoing post‐polymerization, physical aging, and resin network rearrangement become more pronounced over longer durations (4–6 weeks). Clinically, although the absolute changes observed in this study were modest, the presence of significant time‐dependent effects implies that prolonged storage can gradually compromise the fit of the surgical guide. Therefore, minimizing storage time or verifying the fit of guides stored for several weeks may be prudent.

The clinical relevance of the observed RMS magnitudes (approximately 17–51 μm depending on design and condition) is, however, difficult to define precisely, as no universally validated threshold has been established for the maximum acceptable intaglio‐surface deviation of a 3D‐printed surgical guide. Systematic reviews of guided implant surgery have reported acceptable angular and linear deviations of the implant position, with mean coronal and apical deviations of approximately 1 mm under static computer‐aided implant surgery [4], but the chain of error linking intaglio‐surface micro‐deviations to the final implant position is mediated by drilling‐sleeve tolerances, operator handling, and seating verification. The absolute time‐related changes observed here (about 3–5 μm increase over 6 weeks) appear small relative to clinically reported implant positional tolerances, though their precise clinical translation cannot be quantified without a validated threshold. As a practical measure, the seating fit of guides stored for several weeks should be verified before surgical use.

Regarding mechanical properties, specimens stored at 30°C exhibited significantly higher flexural strength compared to those stored at 2°C or 20°C after 6 weeks. This finding aligns with previous studies indicating that elevated temperatures can enhance mechanical performance by promoting additional monomer conversion and network densification. For instance, it has been demonstrated that increasing the post‐curing temperature from 40°C to 80°C significantly improved the flexural strength of 3D‐printed resins [34]. In contrast, another study reported that while high‐temperature treatments (immersion in 100°C water) increased the degree of conversion, extreme conditions such as autoclaving could negatively impact flexural strength, likely due to hydrolytic degradation or excessive thermal stress [35]. In the present study, the relatively mild elevation to 30°C appears to have provided a beneficial thermal environment that facilitated further strengthening without the detrimental effects associated with extreme heat or moisture absorption. Interestingly, flexural modulus was highest at 20°C, significantly exceeding that of the 2°C group, whereas the 30°C group did not differ significantly from the others. This suggests that while strength benefits from warmer storage, stiffness may be optimized within a moderate temperature range. The lower modulus observed at 2°C implies that cold storage may hinder the polymer network from achieving its optimal rigidity, possibly by limiting molecular mobility required for continued cross‐linking during storage. Considered together, the mechanical findings indicate two distinct disadvantages of refrigeration relative to room or warmer storage: lower flexural strength than at 30°C, and lower flexural modulus than at 20°C. By contrast, no measured property favored refrigeration over storage at 20°C or 30°C. These observations therefore reflect material‐level behavior in standardized specimens and are neither supported nor refuted by the dimensional data obtained from the actual surgical guides.

The 3Shape solid‐type guide showed about twice the absolute RMS values (~36–51 μm) of the SMOP open‐frame guide (~17–27 μm) across all conditions. This difference between designs was larger than the temperature or duration effects, but it does not necessarily reflect a true difference in geometric stability. RMS values from best‐fit alignment depend on the spatial extent of the analyzed surface. A small rotational misalignment, which is unavoidable in any best‐fit registration [36], produces local deviations that grow with distance from the alignment fulcrum, so surfaces extending further across the arch include points farther from the fulcrum and thus tend to yield larger RMS values. The same pattern has been reported in the scanner‐accuracy literature, where full‐arch comparisons consistently yield larger RMS values than short‐span comparisons [37]. The position and thickness of the internal connecting bars in the open‐frame design followed the typical clinical workflow of the manufacturer for this case, and both can independently affect the dimensional response. Because such guide‐specific factors as arch extension and bar configuration can themselves influence dimensional stability, the design features of the two guides were not artificially standardized at the design stage in a way that would have departed from clinical practice. Instead, to preserve ecological validity, the two surgical guides were produced by the headquarters design teams of the respective companies in response to the same digitized typodont case, with each team unaware of the design of the other and following the representative clinical workflow of each company. For these reasons, no formal statistical comparison was performed between designs, and the descriptively higher 3Shape RMS values are not necessarily indicative of inferior dimensional stability of the solid‐type design.

It should be noted that the flexural and hardness tests were performed on ISO‐standardized bar and disk specimens rather than on the surgical guides themselves [32]. This approach was used because guide‐level mechanical performance can vary substantially with guide‐specific geometry such as arch shape, span length, connector geometry, and internal supporting bars, which would obscure the intrinsic response of the resin to storage temperature [38]. The trade‐off is that the bar and disk specimens differ from surgical guides in volume, surface‐area‐to‐volume ratio, and load history, so the temperature‐dependent findings characterize the material rather than guide‐level fracture resistance, and direct extrapolation to clinical guide performance would require mechanical testing of full guides.

Vickers hardness did not differ among storage temperatures, suggesting that the surface hardness of this material is relatively stable under the tested conditions and duration. Previous studies have established that surface hardness is primarily determined during the initial fabrication phase, particularly by post‐curing parameters such as temperature and duration, which directly influence the degree of monomer conversion [34, 39]. It has been reported that increasing the post‐curing temperature and time significantly enhances surface hardness by accelerating polymerization and reducing residual monomers [34, 39]. However, once the polymerization network is established, surface hardness appears to be resistant to subsequent thermal exposure compared to bulk mechanical properties. This observation aligns with previous research demonstrating that various post‐fabrication heat treatments, including immersion in boiling water (100°C) and autoclaving, did not significantly alter the Vickers hardness of 3D‐printed resins [35]. Consequently, the storage temperatures evaluated in this study (2°C–30°C), which are much milder than the conditions tested in prior investigations [35], were likely insufficient to induce further physicochemical changes on the specimen surface.

This study has several limitations. First, the observed power for the temperature comparison was low for the 3Shape group (0.085) and modest for the SMOP group (0.300), reflecting the small effect sizes and substantial within‐group variability. The nonsignificant temperature finding should therefore be interpreted as a failure to reject the null hypothesis under the present sample size rather than as evidence of equivalence among temperatures. Second, although the 30‐min equilibration protocol was applied uniformly to minimize transient thermal‐expansion artifacts, the internal temperature of each specimen at the time of scanning was not directly measured. Third, the absolute degree of conversion (DoC) at T0 was not measured directly. If DoC at baseline was sub‐optimal, ongoing post‐polymerization during storage may partly contribute to the observed time‐dependent changes, in particular the flexural strength advantage at 30°C, where mild thermal energy could continue to drive monomer conversion. Future studies should incorporate FTIR‐based DoC measurements at T0 and across the storage period to separate a true storage effect from a cure‐completion effect. Further studies incorporating full‐guide mechanical testing, DoC measurement, and clinically relevant fit/accuracy endpoints are warranted to define optimal storage recommendations for 3D‐printed surgical guides.

## 5 Conclusions

Within the limitations of this in vitro study, the following conclusions were drawn:

1. Storage temperature (2°C–30°C) did not significantly affect intaglio‐surface dimensional stability for either guide design, although the observed power was low. Dimensional deviations increased significantly with storage duration over 6 weeks.
2. In standardized bar and disk specimens (not the guides themselves), flexural strength was higher at 30°C than at 2°C and 20°C, and flexural modulus was higher at 20°C than at 2°C. The dimensional data neither support nor refute these material‐level findings. Vickers hardness was not significantly affected by storage temperature. Refrigeration showed no mechanical advantage; storage at 20°C or 30°C may be preferable for maintaining flexural performance in this material.

## Author Contributions

**Sung‐Jin Kim:** conceptualization, methodology, investigation, data curation, writing – original draft preparation. **Koungjin Park:** conceptualization, methodology, visualization, writing – original draft preparation, writing – reviewing, and editing. **René Daher:** conceptualization, methodology, validation, writing – reviewing, and editing. **Jin‐Soo Ahn:** conceptualization, methodology, resources, writing – reviewing, and editing. **Vincent Fehmer:** conceptualization, methodology, validation, writing – reviewing, and editing. **Jae‐Hyun Lee:** conceptualization, methodology, resources, project administration, supervision, funding acquisition, writing – original draft preparation, writing – reviewing, and editing.

## Funding

This study was supported by grant no 04‐2023‐0125 from the SNUDH Research Fund; the National Research Foundation of Korea (NRF) grant funded by the Korean government (MSIT) (project no. RS‐2025‐24803070).

## Conflicts of Interest

The authors declare no conflicts of interest.

## Supporting information


**Figure S1:** Distributions of intaglio‐surface root mean square (RMS) deviations across storage durations and temperatures, shown as Tukey boxplots (*n* = 10 per condition). Outliers beyond 1.5 × IQR are shown as individual points.

## Data Availability

The data that support the findings of this study are available from the corresponding author upon reasonable request.
